# Additional Effect of Diabetes Mellitus Type 2 on the Risk of Coronary Artery Disease: Role of Serum Adiponectin

**DOI:** 10.5812/ircmj.8742

**Published:** 2014-01-05

**Authors:** Ghorban Mohammadzadeh, Mohammad-Ali Ghaffari

**Affiliations:** 1 Hyperlipidemia Research Center, Department of Biochemistry, Faculty of Medicine, Ahvaz Jundishapur University of Medical Sciences, Ahvaz, IR Iran; 2 Cellular and Molecular Research Center, Department of Biochemistry, Faculty of Medicine, Ahvaz Jundishapur University of Medical Sciences, Ahvaz, IR Iran

**Keywords:** Coronary Artery Disease, Diabetes Mellitus, Type 2, Interleukin-6

## Abstract

**Background:**

Adiponectin, an adipocyte-derived hormone, is implicated in diabetes mellitus type 2 and atherosclerosis. The study was designed to investigate whether serum adiponectin levels in patients with both coronary artery disease (CAD) and diabetes mellitus type 2 (T2DM) are lower than in patients with CAD alone and control subjects.

**Objectives:**

In this present study, we measured serum adiponectin levels in consecutive CAD patients with and without T2DM and investigated whether decreased adiponectin is associated with risk factors of CAD.

**Materials and Methods:**

The study included 198 subjects, 138 patients with CAD (72 of whom had both CAD and T2DM), and 60 control subjects. We measured serum adiponectin, interleukin-6 (IL-6) and insulin by ELISA. In addition, Lipid profile, glucose and anthropometrical measurements were performed in all subjects.

**Results:**

The results revealed significant difference in serum adiponectin levels between patients with CAD+T2DM and patients with CAD alone (3.80 ± 1.52 vs. 5.25 ± 2.35, P = 0.007), between patients with CAD and control (5.25 ± 2.35 vs. 7.04 ± 3.32, P = 0.001), and between patients with CAD + T2DM and control (3.80 ± 1.52 vs. 7.04 ± 3.32, P < 0.001). Serum adiponectin level was significantly higher in women in contrast to men (5.97 ± 3.15 vs. 4.62 ± 2.81 µg/ml, P = 0.002). Serum adiponectin levels were correlated significantly with insulin (r = -0.178, P = 0.013), total cholesterol (r = -0.313, P < 0.001), low density lipoprotein (r = -0.154, P = 0.016), body mass index (r = -0.171, P = 0.016), glucose (r = -0.202, P = 0.006), HOMA-IR (r= -0.251, P = 0.001), and IL-6 levels (r = -0.321, P = 0.001). Adiponectin was correlated positively only with high density lipoprotein (r = 0.389, P < 0.001).

**Conclusions:**

It is speculated that increased insulin resistance and increase in other adipokines such as IL-6 may contribute to the decreased serum levels of adiponectin in patients with both CAD and T2DM.

## 1. Background

Diabetes mellitus type 2 (T2DM) is an established risk factor of cardiovascular disease (CVD), and is associated with a significantly worse prognosis in patients with coronary artery disease (CAD) (1). During the past few years, considerable attention has been paid to the potential role of adipose tissue in the development of vascular complications in diabetes mellitus type 2 (2). The pathophysiological mechanisms linking obesity to CVD are poorly defined; however, adipokines are thought to be involved (3). Although most adipokines appear to promote vascular disease, adiponectin seems to be protective against CVD development. Adiponectin, the major adipocyte secretary protein, is widely known as a beneficial hormone to diabetes and CVD due to its anti-inflammatory, antidiabetic, and antiatherogenic properties (4, 5). Clinically, low levels of adiponectin have been reported in obesity, T2DM, and CAD compared with controls (6, 7). In human, decreased serum adiponectin levels is associated with an increased risk of (CVD), such as a low level of high-density lipoprotein cholesterol, high triglyceride levels, and insulin resistance (8, 9). However, both positive (10-12) and negative (13-15) associations between adiponectin and CAD have been observed. In addition, several (16, 17) but not all (18, 19) epidemiologic studies suggested that reduced plasma adiponectin levels are independent predictors of CAD. Adiponectin levels have also been reported that to be lower in adult men than women (20).

There are several reports about variations of serum adiponectin levels in patients with CAD in different populations. However, the clinical importance of low adiponectin concentrations in T2DM with CAD has not been fully understood, and to our knowledge, serum adiponectin levels and their association with cardiovascular risk factors have not been previously reported in Iranian patients. 

## 2. Objectives

In the present study, we measured serum adiponectin levels in consecutive CAD patients with and without T2DM and investigated whether decreased adiponectin is significantly associated with risk factors of CAD.

## 3. Materials and Methods

### 3.1. Patients Groups

One hundred and ninety-eight Iranian subjects (103 men and 95 women, aged 32 to 85 years) were recruited. Of these 198 subjects, 138 had CAD (72 subjects had both CAD and T2DM). Sixty healthy subjects without T2DM and CAD were selected as control group. Control subjects were characterized by no history of angina and other heart disease. T2DM was defined based on American Diabetes Association (ADA) criteria (the expert committee on the diagnosis and classification of diabetes mellitus 2000). No subjects of T2DM had clinical or laboratory signs of acute infection and none had a history or presence of clinically evidence CVD. They did not receive insulin therapy.

The 138 patients with CAD were consecutively recruited from patients undergoing coronary angiography that had presented with clear evidence of CAD (one or more stenosis greater than 50% in at least one major coronary artery after coronary catheterization and clinical symptoms of angina). The CAD cases were allocated in sub-groups based on the number of significantly stenotic vessels, i.e. subjects with one, two or three affected vessels. Two cardiologists unaware of the impact of their consensus on the study results assessed the grade of coronary stenosis. Patients with renal dysfunction and/or concurrent liver disease were not included. All participants enrolled in the study were Iranians and signed written informed consent. This study was approved by the Ethics Committee of Ahvaz Jundishapur University of Medical Sciences.

### 3.2. Anthropometric Assessments

Anthropometric indices including height and weight were measured while subjects were in the standing position and wearing light clothing without shoes. Body weight was measured in kilograms to the nearest 0.5 kg. Height was measured in centimeters to the nearest 0.5 cm. Body mass index (BMI) was calculated as the body weight in kilogram divided by the square of height in meters (kg/m^2^). These parameters were measured by well-trained dietitians

### 3.3. Blood Sampling and Laboratory Measurements

Biochemical tests were performed on blood samples collected after fasting for at least 12 hours. Venous blood samples for the measurement of fasting serum insulin, adiponectin, Il-6, and lipid profile concentrations were collected into plain and without EDTA-treated tubes. All tubes were centrifuged within several minutes of collections and separated sera stored at -70ºc until assay. For glucose tests, blood was collected into fluorinated tubes and plasma was separated immediately and kept at 4C for up to 48 hours. Fasting plasma glucose was determined by the glucose oxidase method. Total cholesterol, triglycerides, and high- density lipoprotein-cholesterol (HDL-C) were measured by enzymatic methods. Low-density lipoprotein-cholesterol (LDL-C) was estimated indirectly using the Friedewald formula (LDL cholesterol = total cholesterol - HDL cholesterol + 1/5 triglycerides) for subjects with a serum TG concentration of less than 400 mg/ml. Fasting serum insulin was measured by enzyme-linked immunosorbent assay using commercially available human ELISA kit (Q-1-DiaPlus, USA) after the serum samples were thawed at room temperature. This assay has a sensitivity margin of 0. 5 µIU/ml. Intra –and inter-assay coefficients of variation were 6.45 and 6.45%, respectively. Serum IL-6 was measured by enzyme-linked immunosorbent assay (AviBion, human IL-6 ELISA kit, IL06001); intra-assay and inter-assay precisions were ≤ 9.4% and ≤ 8.6%, respectively. Serum adiponectin concentration was measured by enzyme-linked immunosorbent assay using commercially available human adiponectin ELISA kit, (Mediagnost human Adiponectin ELISA kit, E09); intra- and inter-assay coefficients of variation for pooled human serum were 2.35 and 5.70%, respectively. Before assay, quality control and sera were diluted 200 times with dilution buffer, preferably in two steps. Intra- and inter-assay coefficients of variation of all kits used in this study were reported based on the manufacturer data sheet.

### 3.4. Assessments of Insulin Resistance

In each subjects, the degree of insulin resistance was assessed from the fasting glucose and insulin concentrations according to the homeostasis model assessment (HOMA), by the following formula:

HOMA-IR (%) = fasting blood glucose (mg/dl)/18 × fasting insulin (μIU/ml) / 22.5.

### 3.5. Statistical Analysis

All continuous data are expressed as Mean ± SD. Statistics were performed using SPSS for windows version 15 software. Comparison of the mean difference in the adiponectin level between two groups was performed using Student's t-test. Significant differences between groups were compared by one-way analysis of variance (ANOVA) with Tukey's test for post hoc comparisons of each group. Non-normally distributed variables, which were fasting plasma glucose (FPG), systolic blood pressure (SBP), diastolic blood pressure (DBP), HDL-C, HOMA-IR, IL-6, and serum insulin were transformed as natural logarithm before analysis. Pearson correlation coefficients were calculated to evaluate the relationship between serum adiponectin levels and study variables. For all performed tests, P-value < 0.05 was considered as statistically significant.

## 4. Results

**Figure 1.**
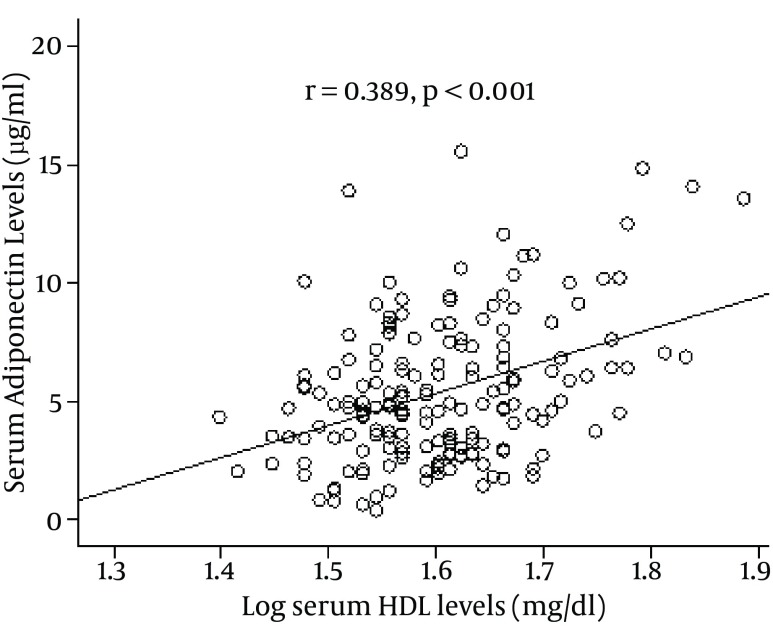
Relationship Between Serum Levels of Adiponectin and Log–transformed of HDL in 198 Study Subjects

**Figure 2.**
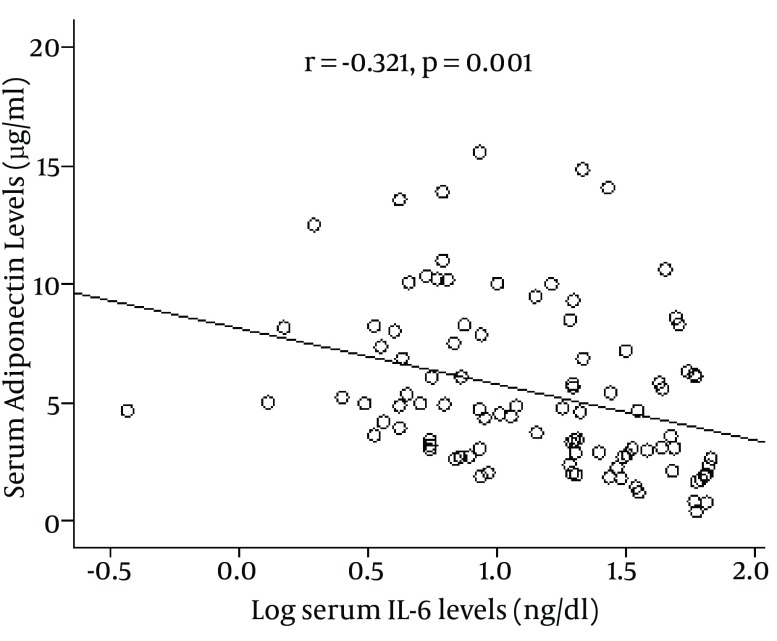
Relationship Between Serum Levels of Adiponectin and Log –transformed of Il-6 in 198 Study Subjects

The anthropometric and biochemical characteristics of the patients and control subjects are presented in Table 1. One hundred and ninety-eight Iranian subjects including 138 CAD (72 of whom had both CAD and T2DM), 66 subjects who had CAD alone without T2DM, and 60 control subjects were enrolled in the study. Patients with both CAD and T2DM had the highest age, SBP, DBP, total cholesterol, triglycerides, FPG, HOMA-IR, IL-6, and insulin levels; ina addition, they had the lowest levels of adiponectin and HDL-cholesterol. The results revealed that there were statistically significant differences in serum adiponectin levels between patients with both CAD and T2DM and patients with CAD alone (3.80 ± 1.52 µg/ml vs. 5.25 ± 2.35 µg/ml, P = 0.007), between patients with CAD and control subjects (5.25 ± 2.35 vs. 7.04 ± 3.32 µg/ml, P = 0.001), and between patients with CAD + T2DM and control subjects (3.80 ± 1.52 µg/ml vs. 7.04 ± 3.32 µg/ml, P < 0.001), as shown in Table 1. Moreover, serum adiponectin level in women was significantly higher than in men (5.97 ± 3.15 vs. 4.62 ± 2.81 μg /ml, P = 0.002; Table 2). Results indicated that there were statistical significant negative correlation between the serum adiponectin level and insulin (r = -0.178, P = 0.013), total cholesterol (r = -0.313, P < 0.001), low density lipoprotein cholesterol (r =-0.154, P = 0.016), body mass index ( r = -0.171, P = 0.016), FPG (r = -0.202, P = 0.006), HOMA-IR (r = -0.251, P = 0.001), SBP (r = -0.173, P = 0.02), DBP (-0.174, P = 0.018), and serum IL-6 levels (r = -0.321, P = 0.001) in all the participants (Table 2). But there were no significant correlation between the serum adiponectin level and age or triglyceride (r = 0.070, P = 0.325; r = -0.145, P = 0.058, respectively). As represented in Figure 1, there was a positive and significant correlation between serum adiponectin levels and serum HDL levels (r = 0.389, P < 0.001) in all participants. Figure 2 graphically illustrates the association of serum adiponectin levels with serum levels of IL-6 in the entire study subjects. It can be seen that there was a negative and significant correlation between serum adiponectin levels and serum levels of IL-6 (r = -0.321, P = 0.001) (Table 3). 

**Table 1.** Anthropometric and Biochemical Characteristics of Patients and Control Subjects in the Study

	CAD + T2DM ^a^	CAD ^a^	Control ^a^	G1 vs. C		G1 vs. G2		G2 vs. C	
				Mean Difference	P Value	Mean Difference	P Value	Mean Difference	P Value
**Age, y**	56.4 ± 9.4	58.0 ± 8.2	50.6 ± 10.85	6.27	0.001	-1.60	0.583	7.87	< 0.001^ b^
**BMI , kg/m** ^**2c**^	26.7 ± 4.2	27.1 ± 4.1	28.4 ± 4.2	-1.67	0.07	-0.42	0.827	-1.24	0.209
**SBP, mmHg ** ^**c**^	2.08 ± 0.06	2.1± 0.05	2.05 ± 0.03	0.023	0.05	-0.023	0.045	0.047	< 0.001^ b^
**DBP , mmHg** ^**c**^	1.8 ± 0.06	1.80 ± 0.07	1.8 ± 0.05	0.012	0.528	-0.018	0.205	0.031	0.017^ b^
**T-cho , mg/dl** ^**c**^	171.2 ± 47.6	178.3 ± 48	165.8 ± 50.8	5.43	0.824	-7.08	0.718	12.51	0.367
**LDL-C , mg/dl** ^**c**^	103.8 ± 44.9	112.4 ± 41	122.6 ± 51.3	-18.84	0.074	-8.59	0.570	-10.25	0.459
**HDL-C , mg/dl** ^**c**^	1.56 ± 0.07	1.57 ± 0.06	1.66 ± 0.08	-0.103	< 0.001	-0.011	0.667	-0.092	< 0.001^ b^
**Tg , mg/dl** ^**c**^	146.2 ± 63.9	178.9 ± 98.4	161.0 ± 74.2	-14.81	0.583	-32.67	0.074	17.85	0.463
**FPG , mg/dl** ^**c**^	2.04 ± 0.1	2.26 ± 0.15	1.95 ± 0.04	0.095	< 0.001	-0.21	< 0.001	0.309	< 0.001^ b^
**Insulin, µIU/m**	1.17 ± 0.4	1.17 ± 0.4	1.05 ± 0.21	0.115	0.237	0.003	1.00	0.115	0.232
**HOMA-IR ** ^**c**^	0.61 ± 0.4	0.75 ± 0.5	0.33 ± 0.15	0.27	0.005	-0.14	0.181	0.42	< 0.001^b^
**IL-6 , ng/ml** ^**c**^	1.19 ± 0.4	1.32 ± 0.4	0.77±0.40	0.41	0.002	-0.13	0.404	0.55	< 0.001^ b^
**AdipoQ , µg/ml** ^**c**^	5.25 ± 2.3	3.80 ± 1.5	7.04 ± 3.38	-1.78	0.001	1.44	0.007	-3.23	< 0.001^b^

^a^ Data are means ± SD.

^b^ P ≤ 0.05 is considered significant; Natural logarithmic transformations were performed before analysis.

^c^ AdipoQ: Adiponectin; BMI: body mass index; C: control; CAD + T2DM: patients with both coronary artery disease and diabetes mellitus type 2; CAD: patients with coronary artery disease; DBP: diastolic blood pressure; FPG: fasting plasma glucose; G1:CAD + T2DM; G2: CAD; HDL-C: High density lipoprotein cholesterol; HOMA-IR: homeostasis model assessment for insulin resistance; IL-6: interleukin 6; LDL-C: Low density lipoprotein cholesterol; mmHg: millimeters of mercury; SBP: systolic blood pressure; T-cho: Total cholesterol; Tg: Triglyceride

**Table 2.** Differences in Serum Adiponectin Levels Between Two Groups

Groups	Serum Adiponectin Levels (µg/ml)	P value
**CAD + T2DM ^a^ (n=72)**	3.80 ± 1.52	0.001 ^b^
**CAD (n=66)** ^**a**^	5.25 ± 2.35	-
**CAD (n= 138)** ^**a**^	4.50 ± 2.54	< 0.001
**Control (n= 60)**	7.04 ± 3.32	-
**Men (n = 103)**	4.62 ± 2.81	0.002
**Women (n=95)**	5.97 ± 3.15	-

^a^ CAD + T2DM: patients with both coronary artery disease and diabetes mellitus type 2; CAD: patients with coronary artery disease

^b^ P values derived by student t-test

**Table 3.** Correlations Between Serum Adiponectin Levels and Cardiovascular Risk Factors

	r	P value
**Age (years)[Table-fn fn6811][Table-fn fn6812]**	0.07	0.325
**BMI (Kg/m2)** ^**c**^	-0.171 ^a^	0.016
**Log SBP (mmHg)** ^**c**^	-0.173 ^a^	0.02
**Log DBP (mmHg)** ^**c**^	-0.174 ^a^	0.018
**Total Cholesterol**	-0.313 ^b^	< 0.001
**LDL-C ** ^**c**^ ** (mg/dl)**	-0.154 ^a^	0.016
**Log HDL-C ** ^**c**^ ** (mg/dl)**	0.389 ^b^	< 0.001
**Triglyceride (mg/dl)**	-0.145	0.058
**Log FPG ** ^**c**^ ** (mg/dl)**	-0.202 ^b^	0.006
**Log Insulin (µIU/ml)**	-0.178 ^a^	0.013
**Log HOMA-IR** ^** c**^	-0.251 ^b^	0.001
**Log IL-6 ** ^**c**^ ** (ng/ml)**	-0.321 ^b^	0.001

^a^ Correlation is Significant at the 0.05 level (2-tailed).

^b^ Correlation is significant at the 0.01 level (2-tailed).

^c^ Abbreviations: BMI: body mass index; DBP: diastolic blood pressure; FPG: fasting plasma glucose; HDL-C: High density lipoprotein cholesterol; HOMA-IR: homeostasis model assessment for insulin resistance; IL-6: interleukin 6; LDL-C: Low density lipoprotein cholesterol; mmHg: millimeters of mercury; SBP: systolic blood pressure; Log: Logarithmic transformation

## 5. Discussion

Clinically, adiponectin appears to serve as a biomarker of CVD. However, as a biologically active molecule, adiponectin appears to protect the vasculature at each stage of atherosclerosis. Atherosclerosis is an inflammatory disease that initially begins with endothelial dysfunction (21). Our study supports the finding of previous studies, demonstrating that lower levels of adiponectin were associated with an increased risk for diabetes and CAD and that serum adiponectin levels are associated with some coronary artery risk factors in patients with CAD. Our main finding is that the serum levels of adiponectin were significantly lower in patients with both CAD and T2DM compared to the CAD alone and healthy controls. Moreover, lower adiponectin level is associated with high risk of developing diabetes mellitus type 2 and coronary artery disease. Decreased levels of serum adiponectin in patients with coronary artery disease (8, 22) and diabetes (7, 23) in our study are consistent with the other studies. Similar to the study by Subhashini et al. (24), we noted that the serum levels of adiponectin are decreased in patients with both CAD and T2DM.

The mechanism leading to decreased plasma adiponectin levels in obesity (20) is not completely understood. Increased adiposity is associated with increased plasma levels of inflammatory markers such as IL-6 (25), hs-CRP (26), and less consistently, TNF-α (27). Especially IL-6 and hs-CRP are established risk markers for cardiovascular events (28). As several cytokines are also produced by adipose tissue, it was postulated that an "adipo-vascular" axis (29) may contribute to the increased risk of cardiovascular events in CAD patients. Previous studies suggested that adiponectin may play a role in the modulation of inflammatory vascular response by inhibiting the expression of adhesion molecules on endothelial cells (30), inhibiting endothelial cells NF-κb signaling (31), and suppressing macrophage function (32). Furthermore, adiponectin knockout mice are prone to increased neointimal formation after vascular injury, and this susceptibility was reversed by adenoviral transfer of adiponectin to these mice (29). Given the anti-inflammatory and vasculoprotective actions of adiponectin and the presentation of obesity as a chronic inflammatory state, the inverse association between decreased serum adiponectin levels and increased serum levels of IL-6 in our study is not surprising.

A strong inverse relationship between serum adiponectin and insulin resistance index, lipid profiles, and blood pressure has been demonstrated in Japanese subjects (33). A large prospective case–control study has demonstrated that high adiponectin concentrations are associated with a lower risk of myocardial infarction (17) suggesting that low adiponectin is not only a marker of cardiovascular risk, but it could also be a causal risk factor. Adenovirus-mediated increase of adiponectin significantly suppresses the progression of atherosclerotic lesions in apoE-deficient mice (34), an animal model that develops hyperlipidemia and vascular lesions similar to human atherosclerosis. In our study, serum levels of adiponectin were significantly and negatively associated with HOMA-IR, total cholesterol, triglyceride, LDL-cholesterol, SBP, and DBP. Moreover, the present study has shown that our findings are similar to other studies in correlation of decreased serum adiponectin levels with BMI and insulin (7). In addition, serum adiponectin level was significantly and positively associated with HDL in our study, suggesting that HDL-C might partly mediate the association between adiponectin and diabetes risk. Adiponectin was associated with substantially higher HDL-C (35), and HDL-c was associated with a borderline-significant reduced risk for diabetes (36). The mechanism by which adiponectin may affect HDL-C levels are largely unknown. Effects of adiponectin on hepatic lipase activity, which is increased in central obesity and insulin resistance, are suspected (37).

We observed a sex differences in serum adiponectin levels in this Iranian subjects. In comparison to men, women had higher levels of serum adiponectin levels. This finding of a sex-based difference in serum adiponectin levels is supported by some authors (37, 38), whereas a few others have failed to observe a sex difference (39, 40). Cnope et al. (37) suggested that the possible explanation for sex differences in adiponectin levels might be due to the different numbers and sizes of fat cells attributed to the different sexes. In addition, Nishizawa et al. (41) indicated that androgen decreased the plasma adiponectin level and the androgen-induced hypoadiponectinemia may be related to a high risk of insulin resistance and atherosclerosis in men. In our study, the serum adiponectin level was lower in men than in women (4.62 ± 2.81 vs. 5.97 ± 3.15 µg/ml). Hypoadiponectinemia in men is thought to be partially responsible for the effect of androgen. However, the total body fat may affect the serum adiponectin levels. Hence, careful interpretation of androgen effect on hypoadiponectinemia in men is necessary .

The current study has some limitations. Because of the cross-sectional nature of the study, this study cannot elucidate mechanisms or determine the direction of causality. Our study included only a small sample size, and larger sample sizes in future studies are needed. Although the small sample size does not enable us to make a definitive conclusion, this is the first study in Iranian subjects with both CAD and T2DM, which revealed that patients with both CAD and T2DM have markedly decreased serum levels of adiponectin compare to patients with CAD alone and control subjects. Thus, T2DM has an additional effect on the risk of CAD, which causes decreasing serum adiponectin levels. In addition, subjects who have markedly decreased levels of adiponectin may be at an increased risk of developing both CAD and T2DM.
